# Long-Term Follow-Up of the Roux-Goldthwait Patellar Stabilization Procedure in a Predominantly Adult Population

**DOI:** 10.7759/cureus.39890

**Published:** 2023-06-02

**Authors:** Inas A Badres, Simon Talbot, John Bartlett

**Affiliations:** 1 Department of Orthopaedic Surgery, Western Health, Melbourne, AUS; 2 Department of Orthopaedic Surgery, Warringal Private Hospital, Melbourne, AUS

**Keywords:** r-g, patellar alta, patellar stabilisation, patellar instability, roux-goldthwait

## Abstract

Background

The Roux-Goldthwait patellar stabilisation (R-G) involves the medial transfer of the distal attachment of the lateral half of the patellar tendon. This paper reviews the long-term results of the R-G in a predominantly adult population.

Methodology

This is a retrospective study looking at patients with recurrent patellar instability who were treated with an R-G technique by a single surgeon over a 36-year period from 1976 to 2012. The primary outcomes measured were further patella instability and further knee surgical procedures.

Results

A total of 202 knees in 170 patients were analysed in this study. Patients between the ages of 9 and 70 years old (average 21 years old) were included in this study. The operative procedure changed during the study period. Initially, patients did not undergo concurrent arthroscopy. Early patients were likely to have additional lateral releases and open medial reefing procedures. More recent patients were more likely to undergo an isolated R-G procedure via a minimally invasive incision. The most common further operative procedure was arthroscopy of the knee for chondral pathology at 13.9%. These were more common early in the study period when patients did not have an initial arthroscopy. Recurrent dislocation was reported at 12.9%, with 5.9% of patients having revision stabilisation surgery, at a mean of 5.58 years (range = 1-15 years) postoperatively.

Conclusions

The R-G procedure is effective in treating recurrent patellar instability in both the paediatric and adult population. It can be performed as an isolated and minimally invasive procedure which is technically simple and has low morbidity.

## Introduction

Patellar instability is a complex condition which usually affects adolescents and young adults. Risk factors include the articular geometry of the knee joint, passive soft tissue restraints, and muscle actions [1]. Multiple factors contribute to recurrent dislocations, and many different techniques have been described to correct the condition.

In 1888, Roux described the medialisation of the patellar tendon for the management of patellar dislocation [2]. Goldthwait in 1895 subsequently described a similar method [3], which was later popularised as the R-G procedure. The procedure has subsequently evolved. Initially, it involved transferring the lateral half of the tendon under the medial half; however, this was thought to cause fat pad scarring and subsequent patellar baja. More recently, in the modified technique, the lateral tendon is transferred superficial to the medial half. It is also used as an isolated procedure instead of in combination with extensive lateral releases and medial reefing procedures.

Other distal realignment procedures that have also been described include the tibial tubercle osteotomy [1] and Hauser [4], Elmslie-Trillat [5], Fulkerson [6], and Maquet [7] procedures. A recent systematic review comparing the different procedures reported a variable rate of recurrence and complications [8]. The aim of our study was to show the long-term outcomes of the R-G procedure by a single surgeon.

This article was previously presented as a meeting abstract at the 2018 Australian Orthopaedic Association Annual Scientific Meeting on October 8, 2018.

## Materials and methods

A total of 202 knees in 170 patients were analysed in this study. Patients between the ages of nine and 70 years old (average 21 years old) were included in this study, with 144 female knees and 58 male knees.

This is a retrospective study examining patients with recurrent patellar instability who were treated with the R-G procedure by a single surgeon over a 36-year period from 1976 to 2012. Ethics approval was obtained from the Institutional Review Board before the commencement of the study. All participants provided written informed consent to participate in the study.

**Figure 1:**
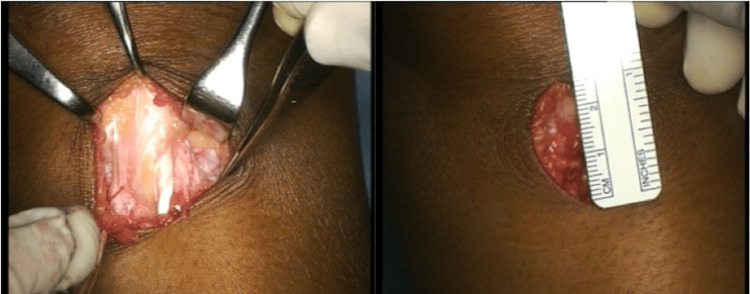
Isolated modified R-G procedure through a minimally invasive 3 cm incision.

The associated surgical procedures evolved over the course of the study. Initially, the R-G was more likely to be combined with an extensive incision to facilitate a lateral release and medial reefing. Routine arthroscopic assessment of the chondral surfaces was introduced in 1996. Towards the end of the series, the R-G was more often an isolated procedure and was frequently performed through a single minimally invasive incision approximately 3-5 cm in length (Figure 1).

**Figure 2:**
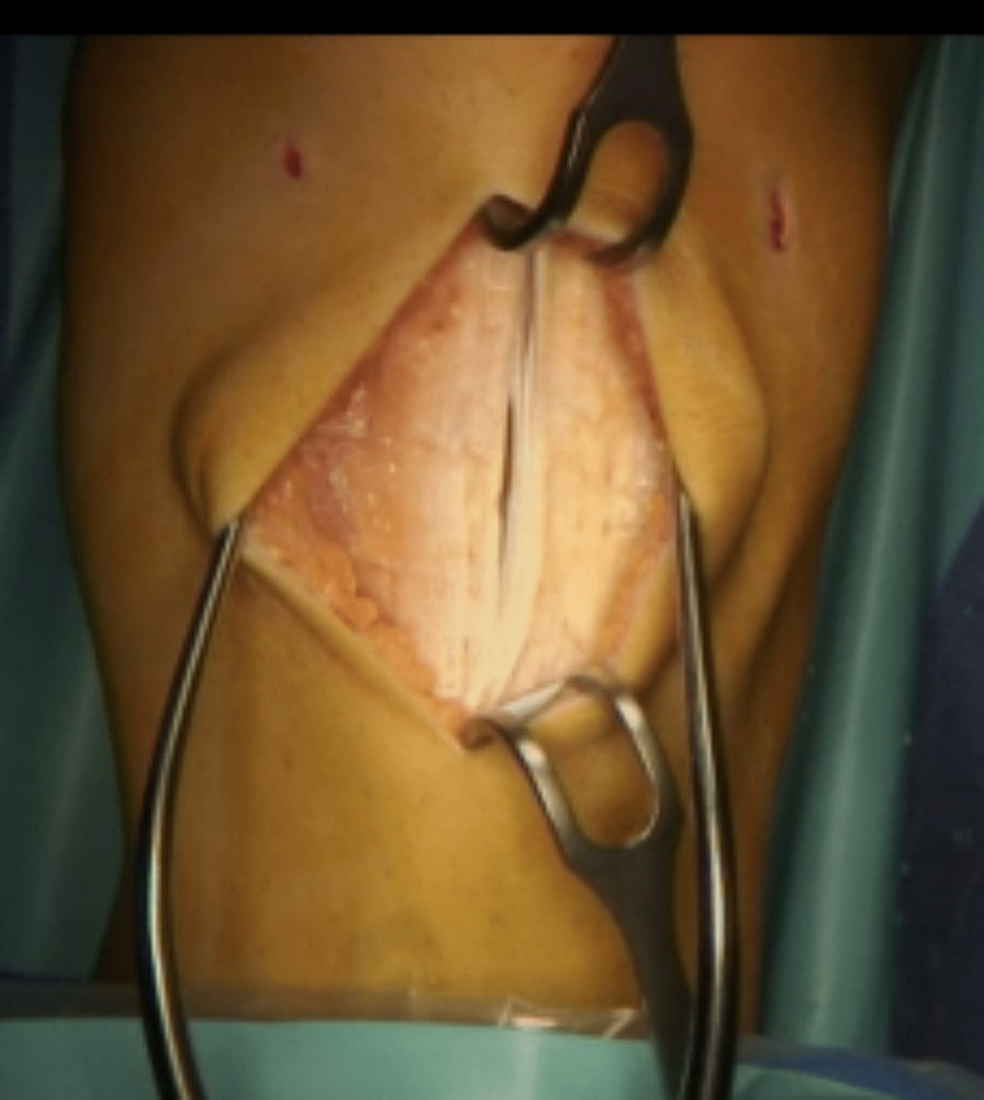
Midline tendon split.

The technique for the R-G part of the surgery remained consistent. A longitudinal incision is made to expose the patellar tendon at the level of the tibial tubercle (Figure 2).

**Figure 3:**
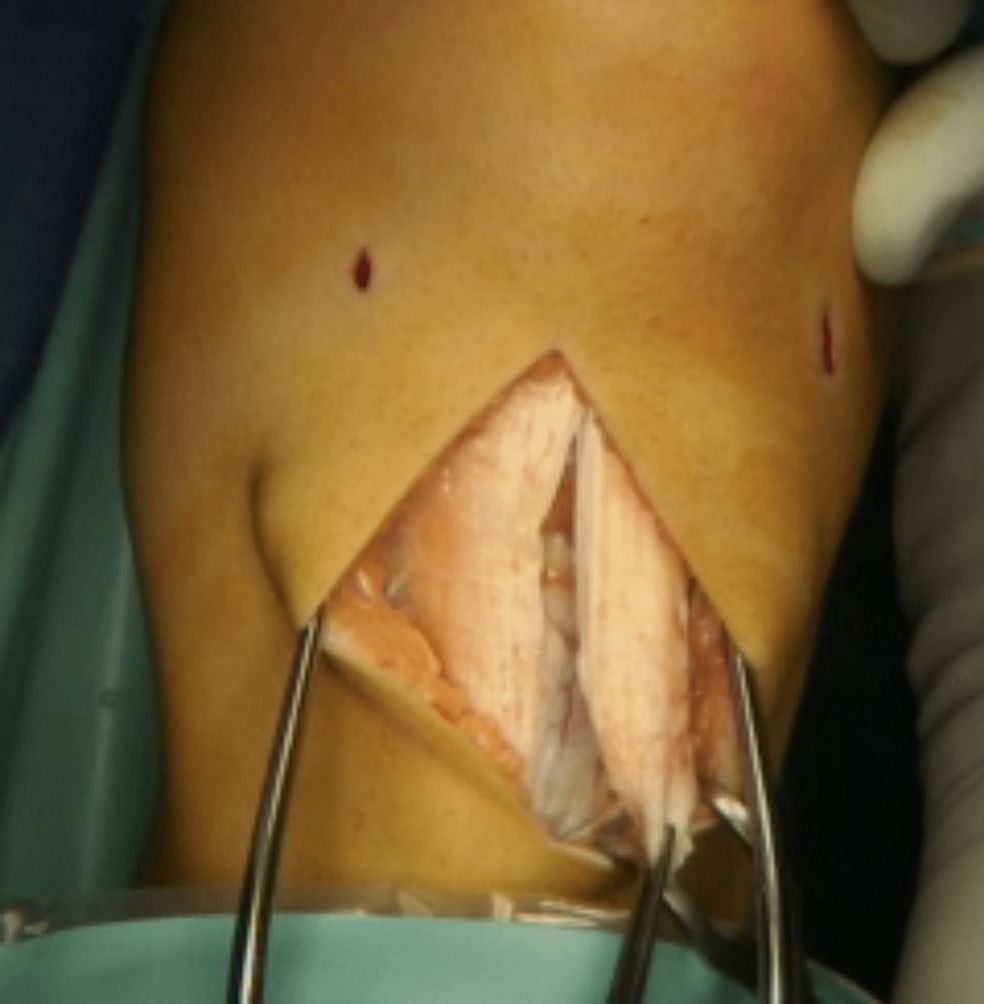
Lateral half of the tendon detached distally.

The patellar tendon is then split along the centre from the tubercle to the distal pole of the patellar and the lateral half is detached distally (Figure 3).

**Figure 4:**
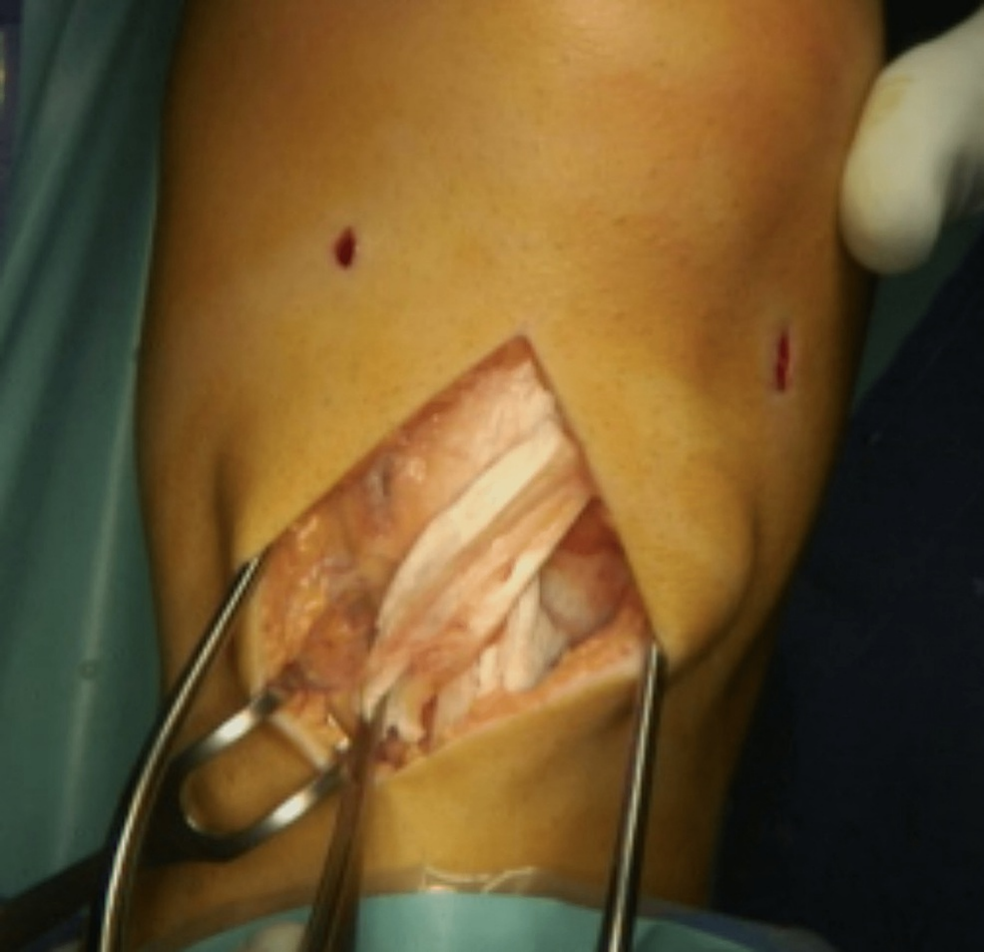
Lateral half of the tendon transferred over the medial half of the tendon and inserted under a periosteal pocket.

A lateral release is performed along the lateral border of the patellar tendon from the tubercle to the distal pole of the patellar. The fat pad is not disturbed. An oblique incision in the periosteum is produced parallel and approximately 1 cm proximal to the pes anserinus tendons. The lateral edge of the pocket is usually adjacent to the medial edge of the tibial tubercle; however, additional medialisation can be achieved as required. A periosteal pocket is developed with a small elevator. The detached tendon is crossed medially superficial to the intact patellar tendon and introduced into the pocket (Figure 4).

**Figure 5:**
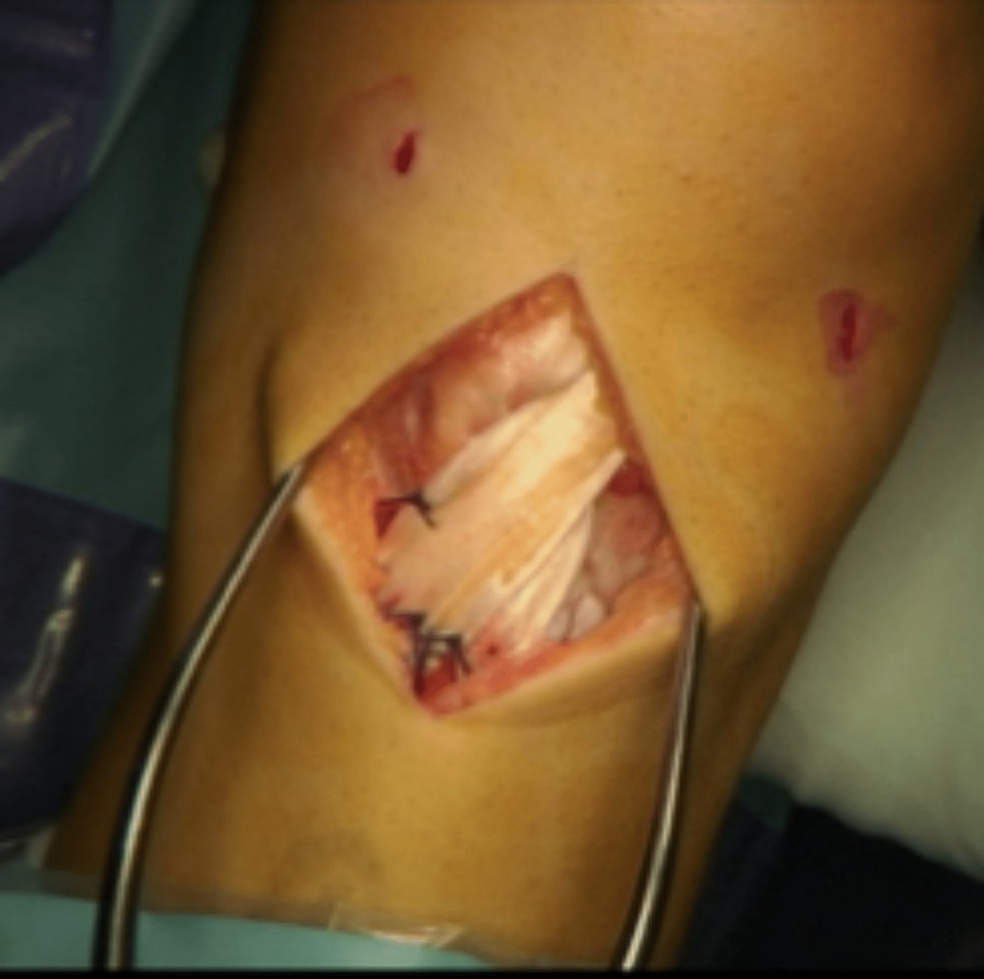
Multiple suture fixation.

Two non-absorbable 2-0 stay sutures on Mayo needles are used to pull the tendon into the pocket and are tied over the periosteum. The tension is adjusted depending on the required reduction in patellar alta based on preoperative radiological assessment. In most cases, between 5 and 10 mm of distalisation is achieved. With the initial stay sutures in position, the position of the patellar is checked arthroscopically. The desired result is for the patellar to centralise in the trochlear groove from 30 degrees of flexion. Additional sutures or bone anchors can be used to further tighten and secure the tendon as required (Figure 5).

To avoid over-tensioning, the knee must be able to flex to 90 degrees at the end of the procedure without stretching the repair or medialisation of the patellar in the groove. The modified technique presented in this study transfers the tendon over the top of the medial half to minimise damage to the fat pad.

In the first five years of the surgeon’s practice, postoperatively, patients were initially managed in a cast in extension for six weeks. However, this changed during the surgeon’s practice to encourage early mobilisation using a continuous passive mobilising machine, with splint protection when weight bearing. The final protocol was to use a hinged-knee brace set at 0-60 degrees for four weeks followed by a full range of motion. Partial weight bearing for four weeks was followed by gradually increasing weight bearing to aim for full weight bearing at six weeks. Static quadriceps exercises were initiated in the first four weeks, and resisted quadriceps exercises were commenced after six weeks. Return to pivoting and contact sports was allowed after three months.

During the study period, the R-G was used for patients with recurrent instability associated with patellar alta and an increased tibial tubercle trochlear distance and varying degrees of trochlear dysplasia. Patients without these features were more likely to receive a medial reefing or medial patellofemoral ligament (MPFL) reconstruction. MPFL reconstruction was first considered in 1989 and subsequently used for patients without patellar alta or severe trochlear dysplasia.

The surgeon kept paper medical records of patients who underwent the R-G procedure. Records were reviewed and patient demographics were recorded onto a database, including date of birth, gender, and date of operation. While searching the medical records, the last follow-up appointment date, further dislocations, and further surgical procedures were also noted.

We then attempted to contact all patients in the study by sending letters. The letter included a questionnaire asking patients if they had further episodes of instability or dislocation and the date of this event. We also asked patients if they had further surgical procedures and the date of this event. Patients were also required to complete patient-recorded outcome measures (PROMs); the Kujala and Oxford Knee Score questionnaire. After two months, patients who did not respond to the letters were contacted by telephone.

**Figure 6:**
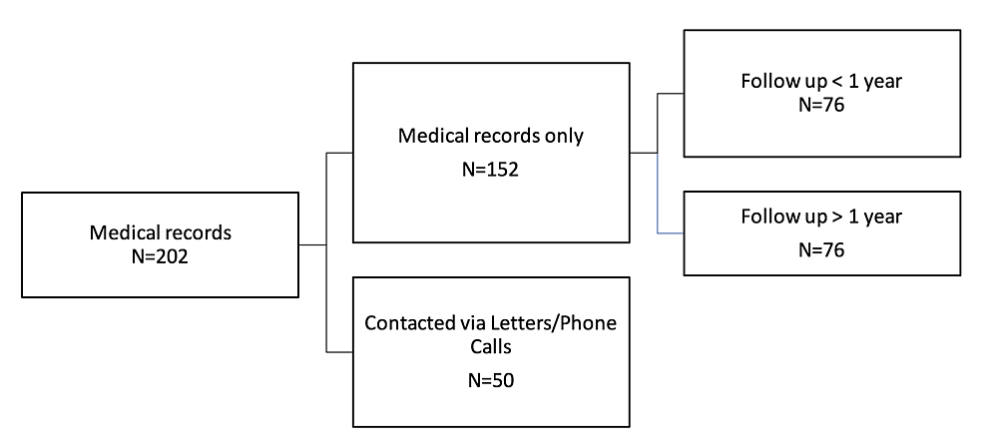
Allocation of patients.

Of the 202 knees included in the study, 76 had a follow-up of less than a year solely from the medical records, 76 had a follow-up of more than a year solely from the medical records, and 50 responded to letters or phone calls (Figure 6).

The primary outcome measures were further symptoms of instability and further surgical procedures. The secondary outcome measures collected were the Kujala and Oxford Knee Score.

Statistical analysis was performed using the SPSS software (IBM Corp. Armonk, NY, USA). Significance was set as p-values <0.05. The chi-square test was used for binomial data.

## Results

**Figure 7:**
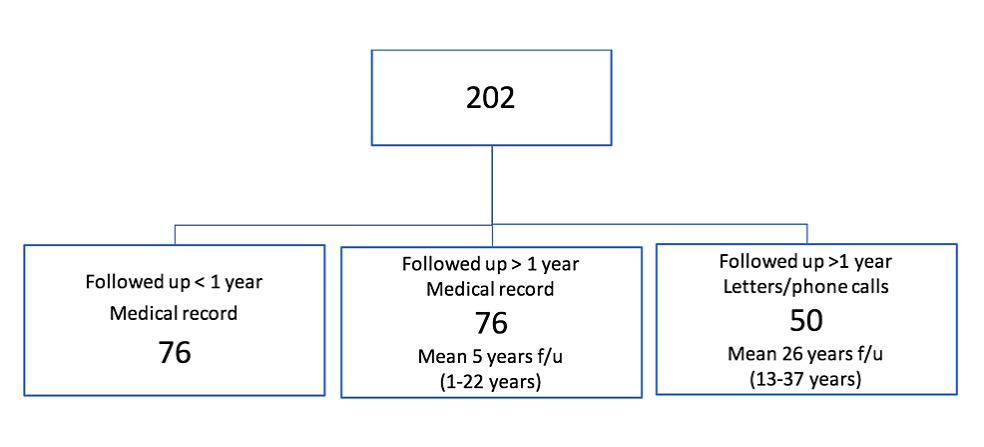
Breakdown of the method and the timeline of follow-up.

During the surgeon’s practice between 1976 and 2012, the R-G procedure was performed on 202 knees in 170 patients. We were able to directly contact 50 patients at an average follow-up of 26 years. These patients completed the Kujala and Oxford Knee Score. The patients who we were unable to follow up directly had a variable follow-up of up to 22 years (Figure 7).

Recurrent dislocations

**Figure 8:**
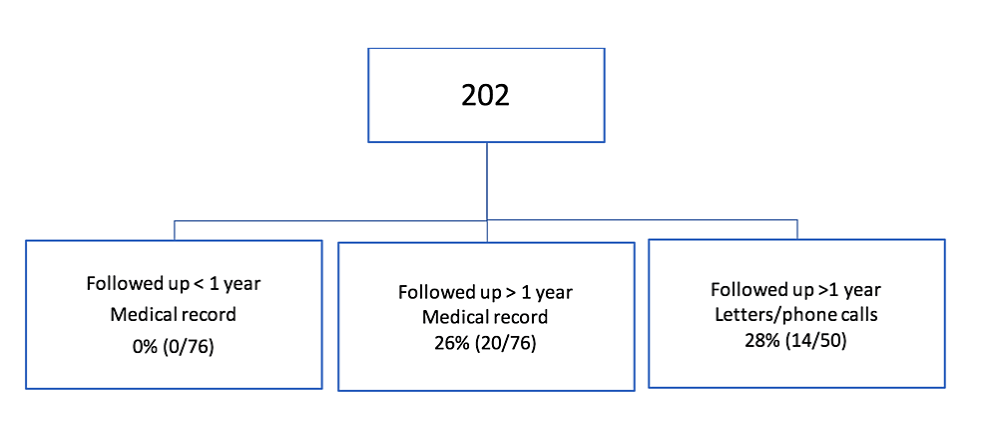
Rate of recurrent instability.

Patients who had a follow-up of more than one year through the medical records reported a 26% rate of further instability symptoms. Patients who were followed up through letters and phone calls had a similar recurrent instability rate of 28% (Figure 8).

Surgical procedures

**Figure 9:**
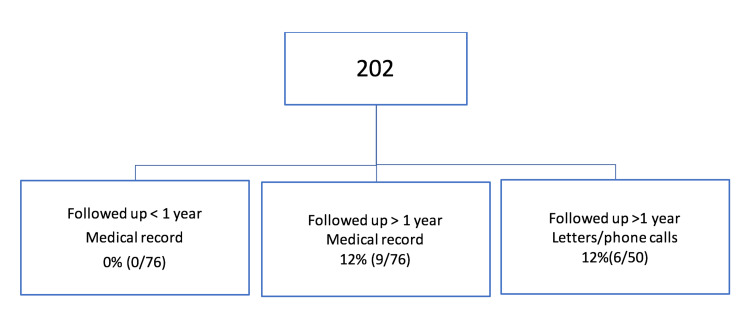
Rate of further surgical procedures.

The group that was followed by a review of records underwent a revision stabilisation procedure at a rate of 12% at an average of five years after the index procedure. The group of 50 that were able to be contacted via letters and phone calls also had a rate of 12%, with the revision stabilisation performed at an average of seven years after the index procedure (Figure 9).

There were 17 revision stabilisation procedures performed in 15 knees, which included 11 revision R-G procedures with lateral release and medial reefing, two tibial tubercle osteotomies, two tibial tubercle osteotomies and MPFL reconstruction, one stabilisation and patellofemoral replacement, and one unspecified stabilisation procedure.

There were 34 additional procedures (32 arthroscopes and two total knee replacements). Most of the arthroscopies were performed within three years of surgery and involved chondroplasty. This could be explained as patients early in the series did not undergo an arthroscopy as part of their initial operation. Since 1996, arthroscopy was incorporated into the additional procedure to deal with chondral pathology and the rate of arthroscopy was reduced.

Secondary outcomes

The 50 patients who were able to be contacted through letters and phone calls completed the Kujala and Oxford Knee Score. The mean Kujala score was 80 (37-100), and the mean Oxford Knee Score was 42 (31-48).

Subgroup analysis

A subgroup analysis was performed to compare the paediatric and adult population results. There were 73 knees that were operated on in patients below the age of 18 (9-17, mean age = 15), and 129 knees that were operated on in patients above the age of 18 (18-70, mean age = 25). In the paediatric population, there was a 27% rate of further dislocations, while in the adult population, there was a 10% rate of further dislocations (p = 0.0014). There was no difference in the rate of further revision stabilisation procedures between adult and paediatric groups, with 11% in the paediatric population and 8% in the adult population (p = 0.4422) (Table 1).

**Table 1:** Patient demographics and outcomes. F = female; M = male; R = right; L = left

	Paediatric	Adult	P-value
n	73	129	
Age	9–17 (mean 15)	18–70 (mean 25)	
Gender	F:M (%) 77:23	F:M (%) 68:32	0.02
Side	R:L 40:60	R:L 50:50	0.14
Further dislocations	27%	10%	0.0014
Further revision stabilisation procedures	11%	8%	0.4422

## Discussion

Our study showed an increased rate of dislocation in the paediatric population at a rate of 27% compared to a rate of 10% in the adult population. There was also a higher proportion of females in our study which is similar to other studies as females have a higher risk of recurrent patella dislocations [9,10]. The increased rate of dislocation in the paediatric population could be attributed to the fact that patellar instability occurs predominantly in patients between the ages of 10 and 17 years [9,11-14]. This could also be attributed to the higher percentage of females in the paediatric cohort compared to the adult cohort of our study.

Roux first described a distal realignment procedure in 1888 to manage recurrent patellar dislocation, which included medial retinacular plication and lateral release in combination with medial transfer of the lateral half of the patellar tendon [2,15,16].

There have been many studies which have looked into the effectiveness of the R-G procedure in skeletally immature patients [10,17-19]. Vahasarja et al. in 1995 studied 57 operations comparing lateral release alone, lateral release with medial reefing, and the R-G procedure. Of the 57 knees in their study, only three were treated with the R-G solely. Furthermore, 13 of the remaining 54 knees required further surgical procedures, 10 of which subsequently underwent an R-G [10]. Marsh et al. in 2006 studied 30 knees of skeletally immature patients and revealed an 87% excellent result using Insall’s criteria [19,20]. In 2018, Ruzzini et al. studied the R-G procedure in 23 knees of skeletally immature Down syndrome patients and showed an improvement in the range of motion in these patients [17].

There are not many studies examining the effectiveness of the R-G procedure in the adult population. Even when such studies exist, the number of participants in the study is low. Our paper aims to fill the gap in the literature. Fondren et al. in 1985 examined 47 knees in a combined paediatric and adult population which showed excellent results in 12 knees and good in 31 knees using Insall’s criteria [20,21]. Before this, Chrisman et al. in 1979 examined 87 knees in patients aged between 9 and 43 years comparing the Hauser and R-G procedure and showed that 28 out of 40 had excellent results in the R-G patients when using the Grana and O’Donoghue rating system [15,22].

The issues we identified when trying to compare our results with previous studies were the lack of consistency with measuring endpoints and the success of the procedure, with most studies using subjective measures. A systematic review which was performed recently attempted to address this by looking at studies which specifically examined outcome measures and range of motion, recurrence of instability, complications, and osteoarthritis [8]. The literature which examined 21 studies looked to compare the effectiveness of the Elmslie-Trillat, Maquet, Fulkerson, R-G, and other distal realignment procedures in the management of patellar dislocations [8]. The study had a mean follow-up of 9.8 years and showed variable results with a 0-50% rate of further instability and a 0-50% rate of additional and revision surgery [8]. The mean Kujala scores were also highly variable ranging from 68 to 86 [8]. In comparison, our study had a much longer follow-up of a mean of 26 years. Our study which showed a 26-28% rate of further instability and a revision rate of 12% was comparable to the current literature. Our mean Kujala score was 80 which is also comparable to the literature.

The surgical indication for the R-G procedure is the need for a distal realignment procedure to improve patellar stability. It also has the added benefit of being able to reduce the degree of patellar alta and the length of the patellar tendon. Therefore, it is largely indicated in patients with patellar alta and lateralisation of the tibial tubercle. In patients with mild-to-moderate trochlear dysplasia, this distalisation of the patellar brings it into the deeper distal section of the groove. As a distal realignment procedure, the R-G can be considered an alternative to tibial tubercle osteotomy (TTO) as TTOs have a high rate of postoperative complications such as non-union, tubercle or tibial shaft fracture, and the need for further metalware removal [23-25].

MPFL reconstruction is another common operation for recurrent patellar dislocation. It has been shown to have excellent results in some studies but relatively high rates of recurrent instability and complications in others [26-28]. Recurrent instability is quoted between 5% and 30%, with complications including patellar fracture and a return to theatre for manipulations to address the decreased range of motion and removal of symptomatic hardware [26-29]. In patients with normal or relatively normal anatomy and severe medial soft tissue disruption, MPFL reconstruction would appear to be an excellent choice. In the current study, MPFL was not considered in patients before 1989, and there may have been patients in this early phase who would have been better treated with MPFL reconstruction. However, in patients with trochlear dysplasia, patellar alta, and a lateralised tibial tubercle, the reconstruction of the medial stabilisers is at best recreating the situation which failed initially. In these cases, addressing the distal alignment of the extensor mechanism with either a TTO or R-G procedure seems sensible.

The need for secondary arthroscopy in this series was very high. These arthroscopies were largely done to address chondral issues in the first one to two years after the initial surgery. The rate of these decreased markedly after arthroscopy was routinely included in the index procedure from 1996. This suggests that many of the chondral issues existed, but were not identified, at the time of the index procedure. Chondral injury and progressive degeneration have been raised as possible risks of the R-G due to increased patellar reaction forces associated with medialisation and distalisation of the patellar relative to the trochlear groove. In a study that compared the R-G procedure to ligament reconstruction, both showed comparable grade II to grade IV chondral lesions on MRI at a median 10-year follow-up [30].

The R-G procedure can also be tailored to patients with more normal anatomy with the absence of patellar alta.

The strength of this study is that it is a large cohort study which includes 202 knees. It also has a long follow-up period of up to 36 years in the course of a single surgeon’s practice.

The limitations of this study are the low rate of direct follow-up of patients and the heterogeneity of the surgical procedures performed. Due to the long-term retrospective nature of the study, the only contact details for most patients were addresses and home phone numbers. Therefore, we were unable to contact the majority of patients for direct follow-up. However, given the similar results of a revision rate of 12% in both the direct follow-up cohort (six of 50 at a mean of 26 years) and medical records cohort (nine of 76 at a mean of five years), it would suggest that the revision rate of 12% is reasonably robust.

Due to the nature of the retrospective study, we acknowledge that there are further limitations to the data collected including preoperative PROMS and preoperative and postoperative range of motion.

The evolution of the associated surgical techniques over the 36 years of the study period makes it difficult to isolate the importance of the R-G procedure. The technique for the modified R-G procedure was very consistent during this time period. The steady reduction in lateral releases and medial reefing procedures during the period leads to an increasing reliance on distal realignment to achieve stability.

Further prospective studies are required to further assess the effectiveness of the isolated, modified R-G procedure in the adult population.

## Conclusions

The modified R-G procedure is effective in treating recurrent patellar instability in both the paediatric and adult population. It can be performed as an isolated and minimally invasive procedure which is technically simple and has low morbidity, and it should be considered in patients with patellar instability, which is associated with patellar alta and a lateralised tibial tubercle.
